# Supplementary material for: Selective hematopoietic stem cell ablation using CD117-antibody-drug-conjugates enables safe and effective transplantation with immunity preservation
Source: Nat Commun. 2019 Feb 6;10:617. doi: 10.1038/s41467-018-08201-x (PMC6365495; doi:10.1038/s41467-018-08201-x)
Supplement: Supplementary file 1 — Supplementary Information [file 41467_2018_8201_MOESM1_ESM.pdf]

Selective Hematopoietic Stem Cell Ablation using CD117-Antibody-Drug-Conjugates Enables Safe and Effective Transplantation with Preservation of Immunity

Czechowicz et al.

Supplementary Information

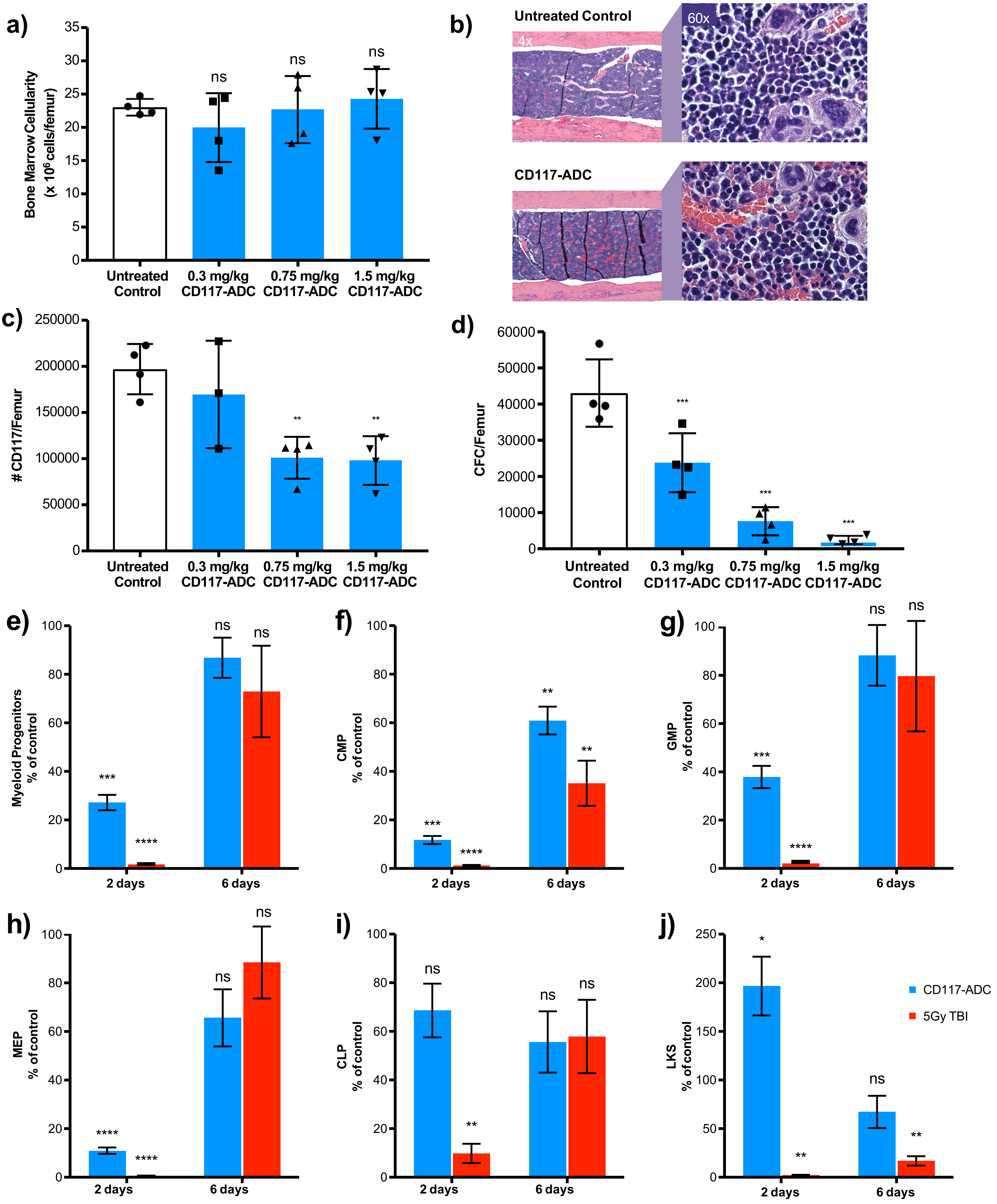

Supplementary Figure 1. One-time, intravenous treatment with CD117-ADC spares many hematopoietic progenitors.

a) CD117– ADC spares most bone marrow cells with intact cellularity 8 days post treatment. **b)** Histologically normal bone marrow H&E staining 8 days post CD117–ADC treatment. **c)** Despite robust HSC depletion, many CD117-expressing hematopoietic progenitor cells present 8 days post CD117-ADC treatment. **d)** CD117– ADC additionally decreases HSPC *in vitro* activity as assessed by CFC assays established with WBM from mice treated 8 days prior but some activity remains. **e-j)** At early time-points, CD117-ADC affects various myeloid hematopoietic progenitors (e), common myeloid progenitors (f), granulocyte-monocyte progenitors (g), megakaryocyte-erythroid progenitors (h), common lymphoid progenitors (i), and HSPC (Lin-cKit+Sca1+) cells (j) but less so than 5Gy total body irradiation, and most progenitors rebound to near normal levels by 6 days post CD117-ADC treatment. Statistics calculated using unpaired *t* test. Data represent mean ± SEM. (*n* = 3-4 mice/group, assayed individually); all data points significant as indicated vs. untreated controls (****P* < 0.001).

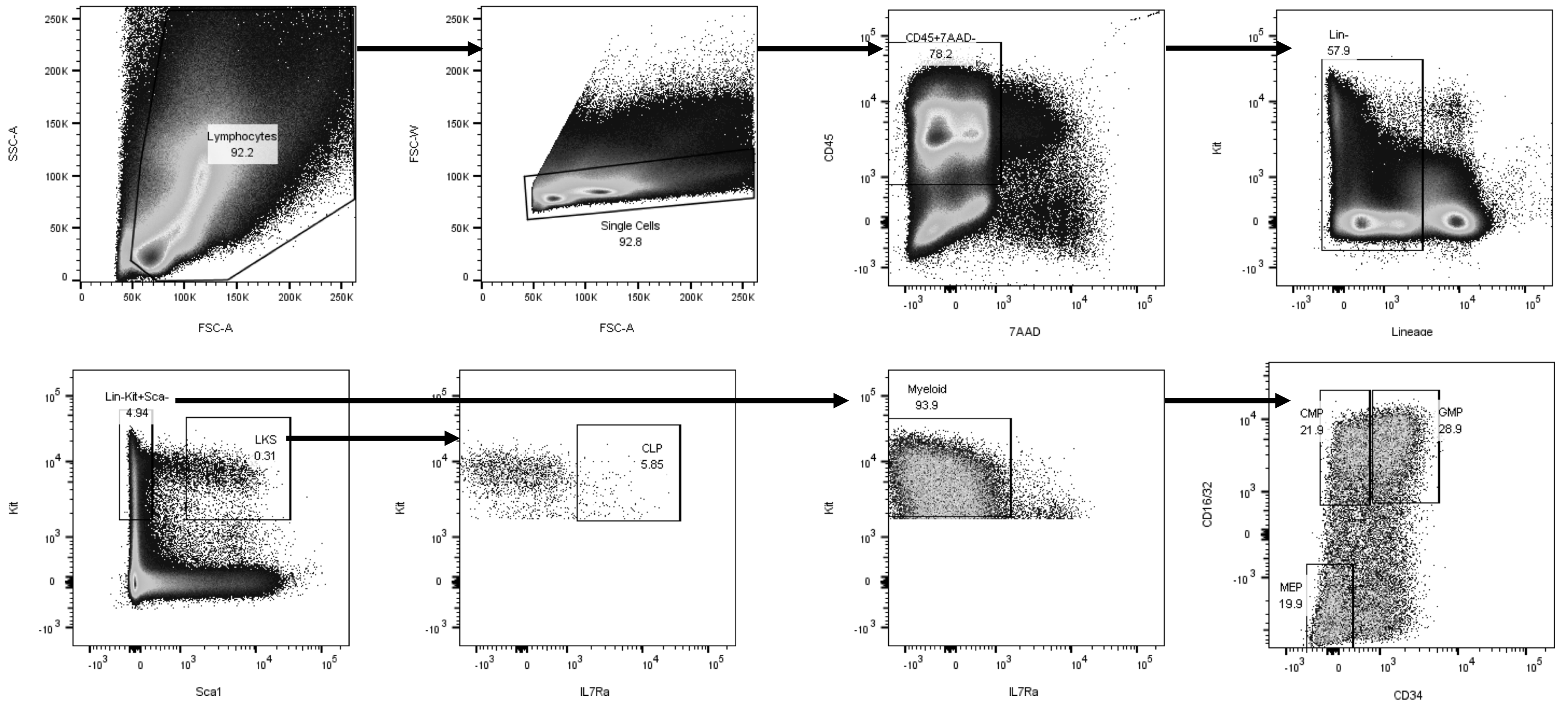

Supplementary Figure 2. Representative flow cytometry plot of bone marrow hematopoietic progenitor cell assessment.
Bone marrow assessment of untreated (shown), CD117-ADC and 5Gy TBI treated animals was performed via standard gating scheme to determine myeloid hematopoietic progenitor, common myeloid progenitor (CMP), granulocyte-monocyte progenitor (GMP), megakaryocyte-erythrocyte progenitor (MEP), common lymphoid progenitor (CLP) and hematopoietic stem/progenitor, Lin-Kit+Sca- (LKS) cell composition.

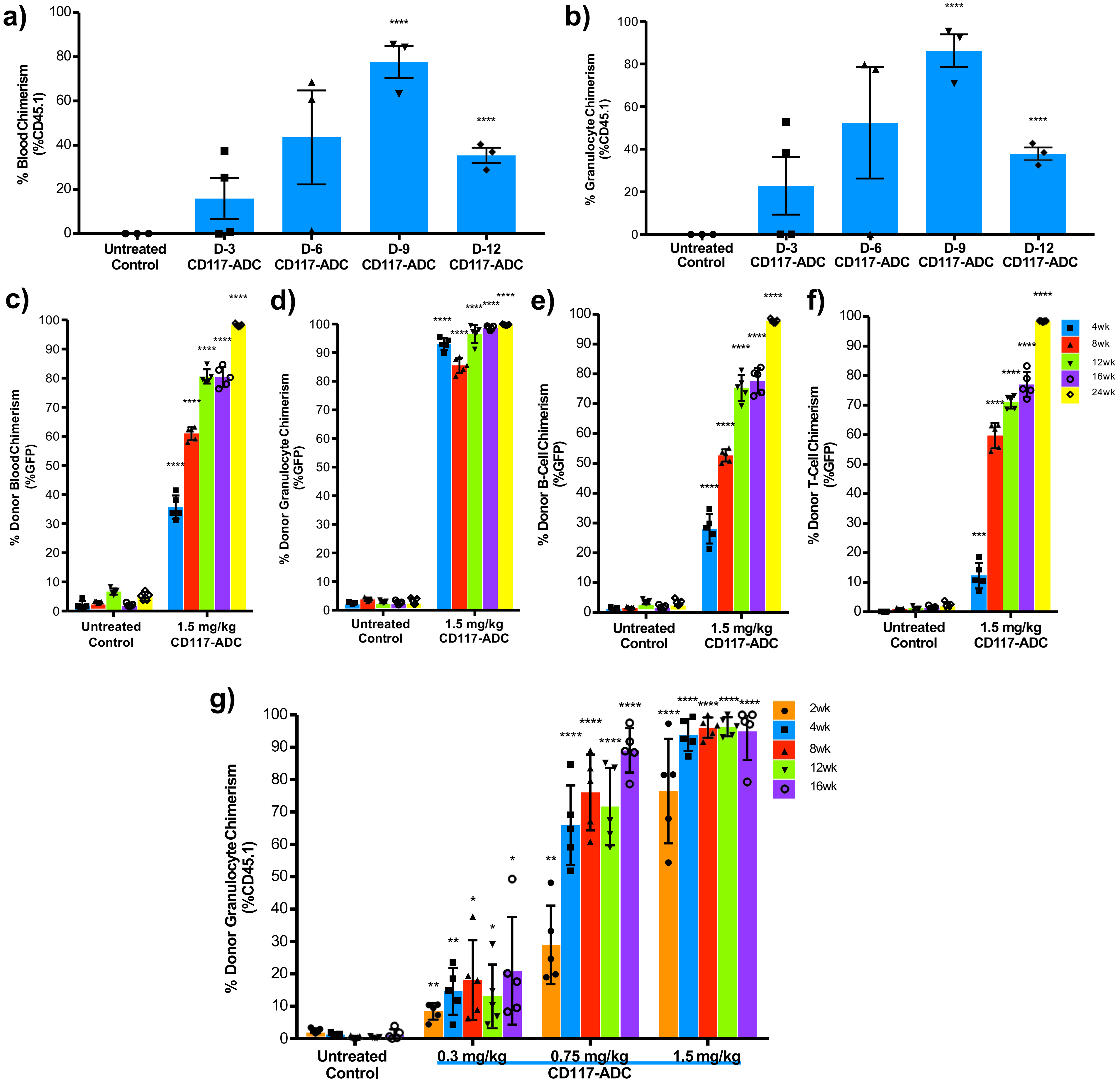

Supplementary Figure 3. CD117-ADC conditioning effectively enhances donor murine WBM engraftment, with wide transplantation window.
a,b) Enhanced and persistent donor peripheral blood (a) and granulocyte engraftment (b) observed 20 weeks post CD117-ADC treatment and subsequent transplantation of 10x106 CD45.1+ donor whole bone marrow cells regardless of tested treatment time-point, however highest engraftment observed with pre-treatment 8-9 days prior to transplantation. c-f) Similarly enhanced and persistent donor WBM engraftment observed with CD117-ADC treatment and subsequent transplantation of 10x106 GFP+ donor whole bone marrow cells 8 days after with robust multi-lineage donor engraftment of >97% in all peripheral blood (c), granulocytes (d), B-cells (e) and T-cells (f). g) Significant and increasing donor engraftment observed with 0.3 mg/kg to 1.5 mg/kg CD117-ADC pre-treatment and subsequent transplantation of 10x106 CD45.1+ donor whole bone marrow cells. Data represent mean ± SEM (n = 3-5 mice/group, assayed individually). Statistics calculated using unpaired t test; all data points significant as indicated vs. untreated control (*P < 0.05; **P < 0.01; ***P < 0.001; ****P < 0.001).

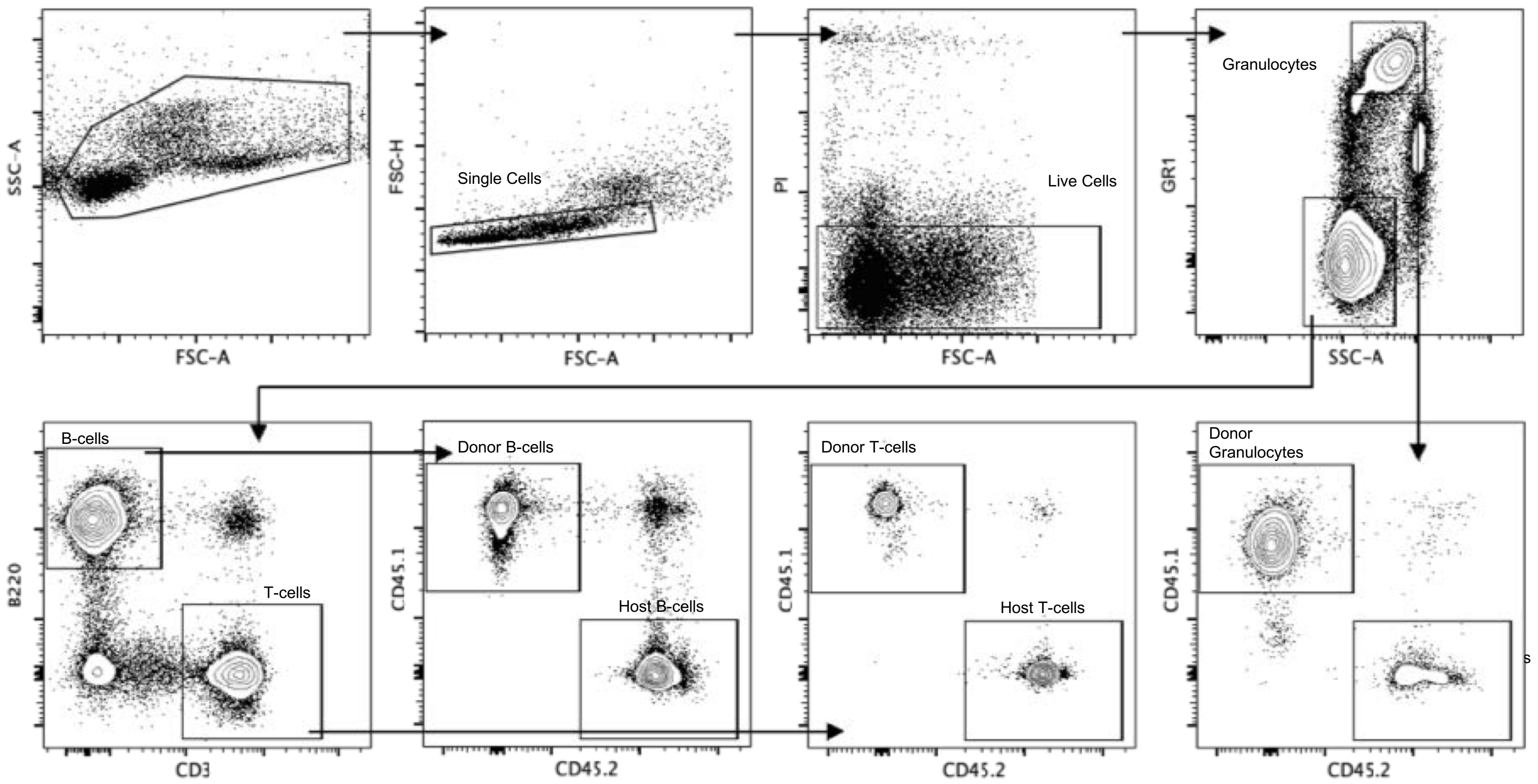

Supplementary Figure 4. Representative flow cytometry plot of peripheral blood hematopoietic cell subset assessment.
Peripheral blood assessment of transplanted recipient mice post CD117-ADC (shown) or other conditioning treatments was performed via standard gating scheme to assess for donor reconstitution of B-cells, T-cells and granulocytes.

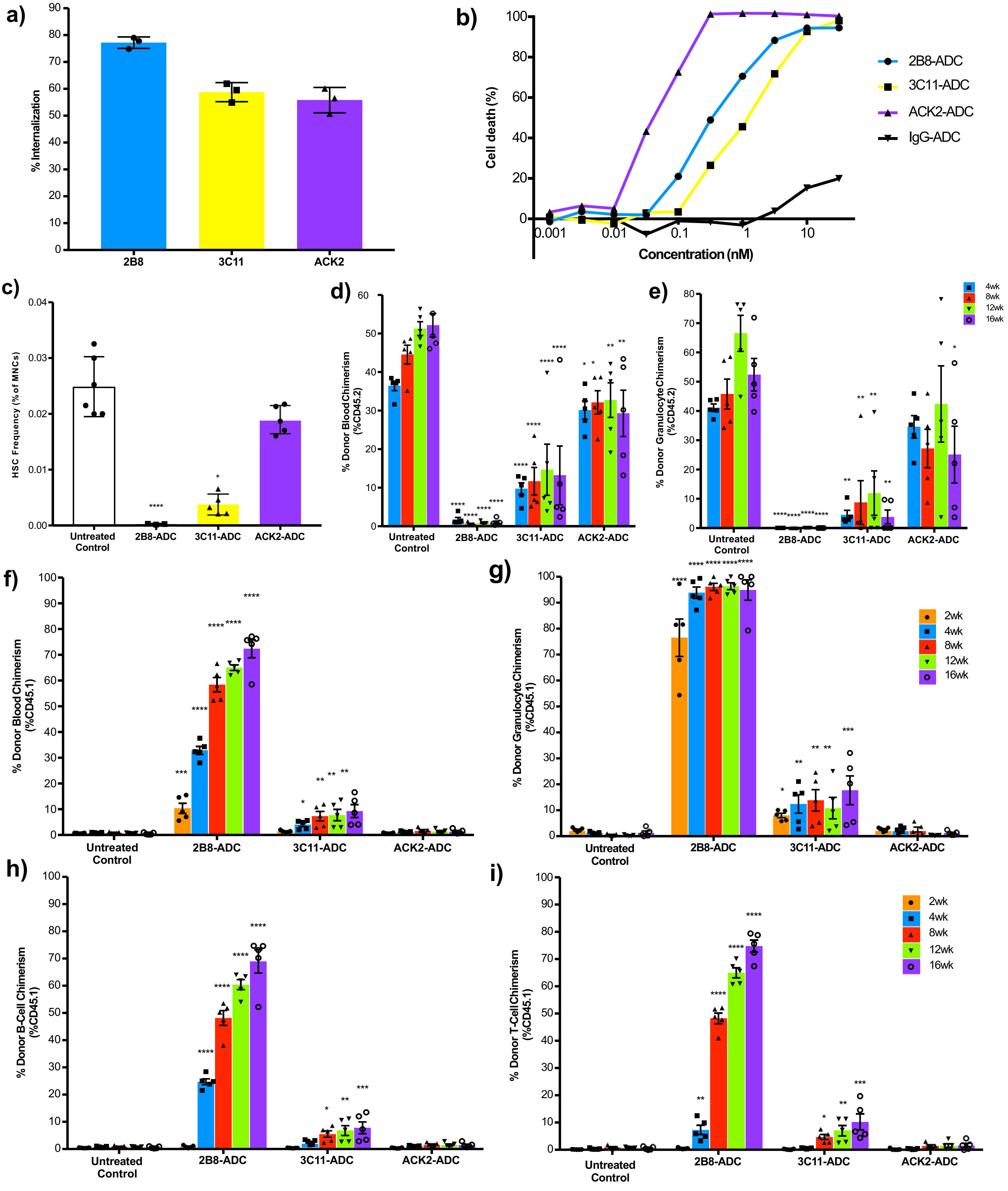

Supplementary Figure 5. Multiple CD117-ADCs can be used to effectively deplete HSCs *in vitro* and *in vivo* and enable enhanced donor WBM transplantation, with most potency observed with 2B8-ADC.

a) Internalization of various antibodies by CD117-expressing EML cells with highest internalization observed with anti-CD117 mAb 2B8 clone. (4hr incubation, mean ± SEM of n=6 replicates). **b)** *In vitro* cell death of EML cells observed post treatment of all CD117-ADCs (72hr incubation, mean ± SEM of n=3 replicates). **c)** Phenotypic depletion of HSCs as assessed by flow cytometry (Lin−cKit+Sca1+CD48−CD150+) 8 days after IV administration of all CD117–ADCs, with most activity noted with 2B8 clone bound to saporin toxin, as compared to 3C11 and ACK2 clones. **d,e)** Functional HSC depletion of all CD117-ADCs confirmed by lack of long-term, 16-week, total donor peripheral (d) and granulocyte (e) chimerism in competitive transplantation post treatment of WBM from CD117-ADC treated animals. **f-i)** Multiple CD117-ADCs effectively enable enhanced multi-lineage donor WBM engraftment, enhanced engraftment in total donor peripheral blood chimerism (f), donor granulocyte chimerism (g), donor B-cell chimerism (h), and donor T-cell chimerism (i), with most potency observed with 2B8-ADC which also resulted in most robust HSC depletion. Data represent mean ± SEM (*n* = 5 mice/group, assayed individually); Statistics calculated using unpaired *t* test; all data points significant as indicated vs. untreated control (**P* < 0.05; ***P* < 0.01; ****P* < 0.001; *****P* < 0.0001).

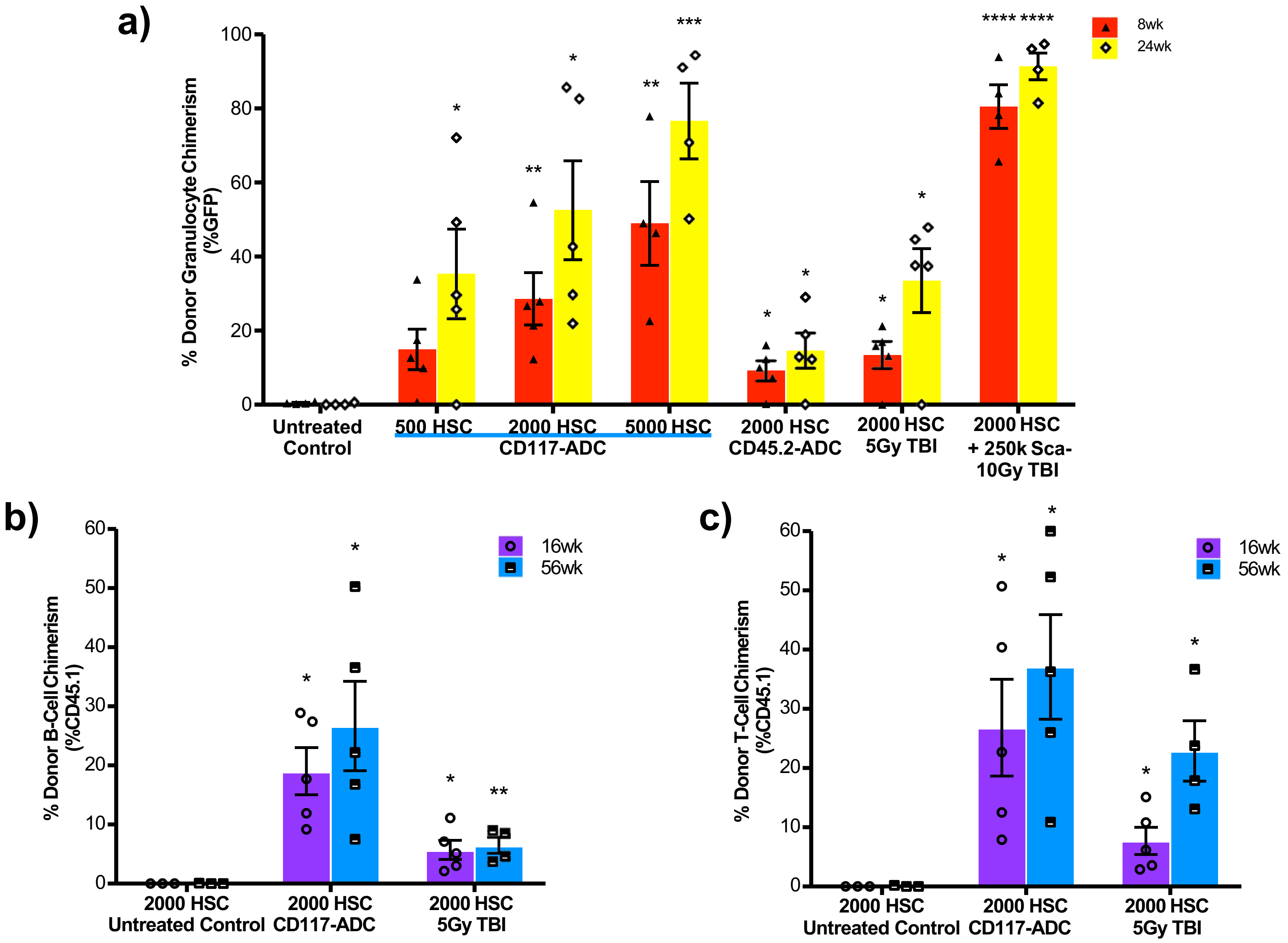

Supplementary Figure 6. CD117-ADC conditioning enables robust immune reconstitution post purified HSC transplantation.
a) CD117-ADC pre-treatment 8 days before infusion of FACS-purified GFP+ donor HSCs leads to robust donor granulocyte chimerism, with increasing engraftment with increased HSC cell dose. **b,c)** Enhanced donor B-cell (b) and T-cell (c) engraftment at late time points post transplantation of purified HSCs into CD117-ADC conditioned animals as compared to 5Gy TBI controls. Data represent mean ± SEM (*n* = 3-5 mice/group, assayed individually); Statistics calculated using unpaired *t* test; all data points significant as indicated vs. untreated control (**P* < 0.05; ***P* < 0.01; ****P* < 0.001; *****P* < 0.0001).

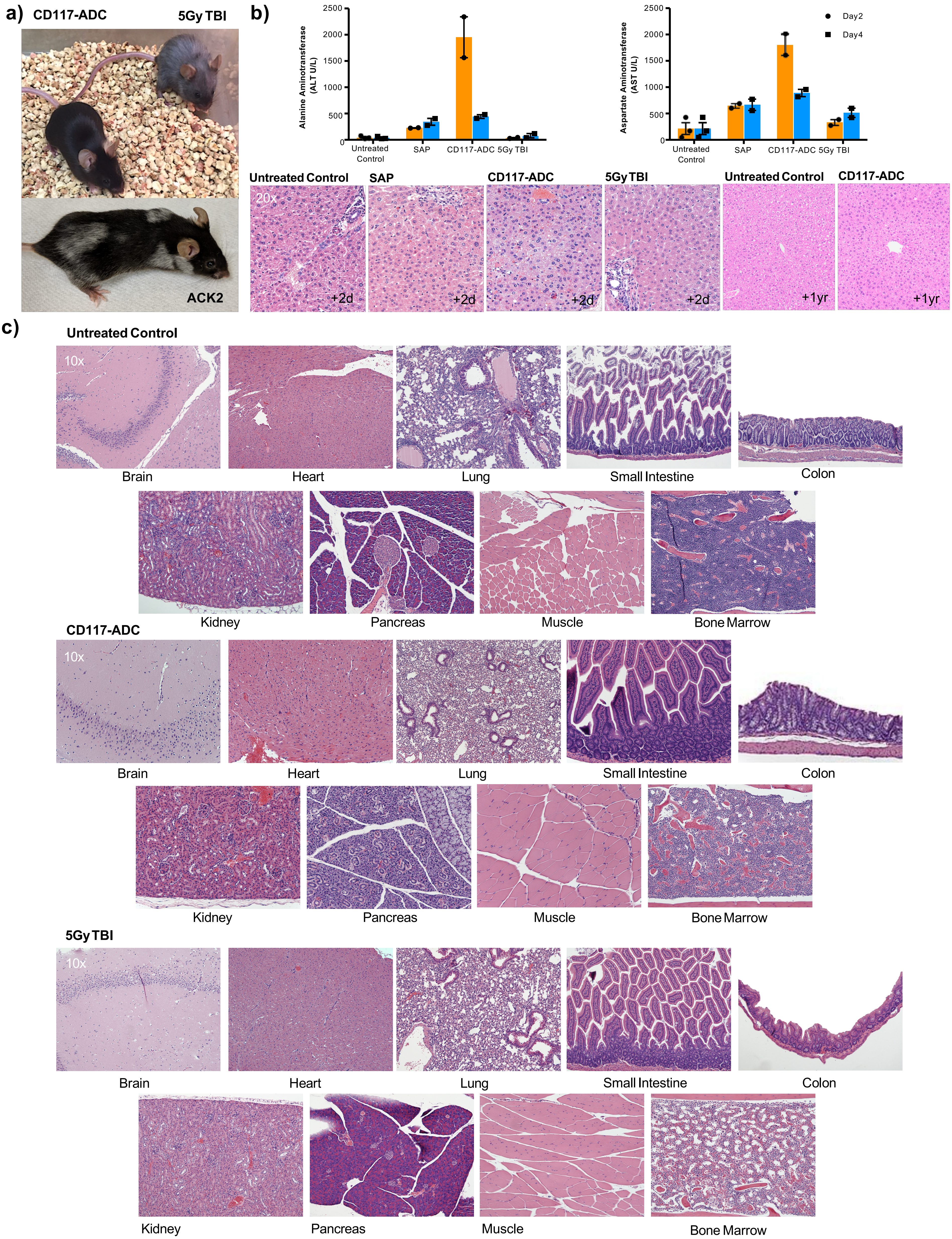

Supplementary Figure 7. At therapeutic doses, CD117-ADC is grossly non-toxic apart from transiently elevated transaminases.
a) CD117-ADC treated animals appear healthy with full coat color as compared to post 5Gy TBI or ACK2 even 24 weeks post treatment. **b)** Saporin toxin alone causes minor liver toxicity with elevation of transaminases alanine aminotransferase (ALT) and aspartate aminotransferase (AST), which is further transiently elevated post CD117-ADC treatment with resulting intermittent apoptotic hepatocytes which resolves with long-term normal liver histology. **c)** All other organs assessed looked grossly normal by H&E histology 2 days post CD117-ADC treatment including brain, heart, lung, small intestine, colon, kidney, pancreas, muscle and bone marrow, unlike 5Gy TBI. *n* = 2-3 mice/group, assayed individually.

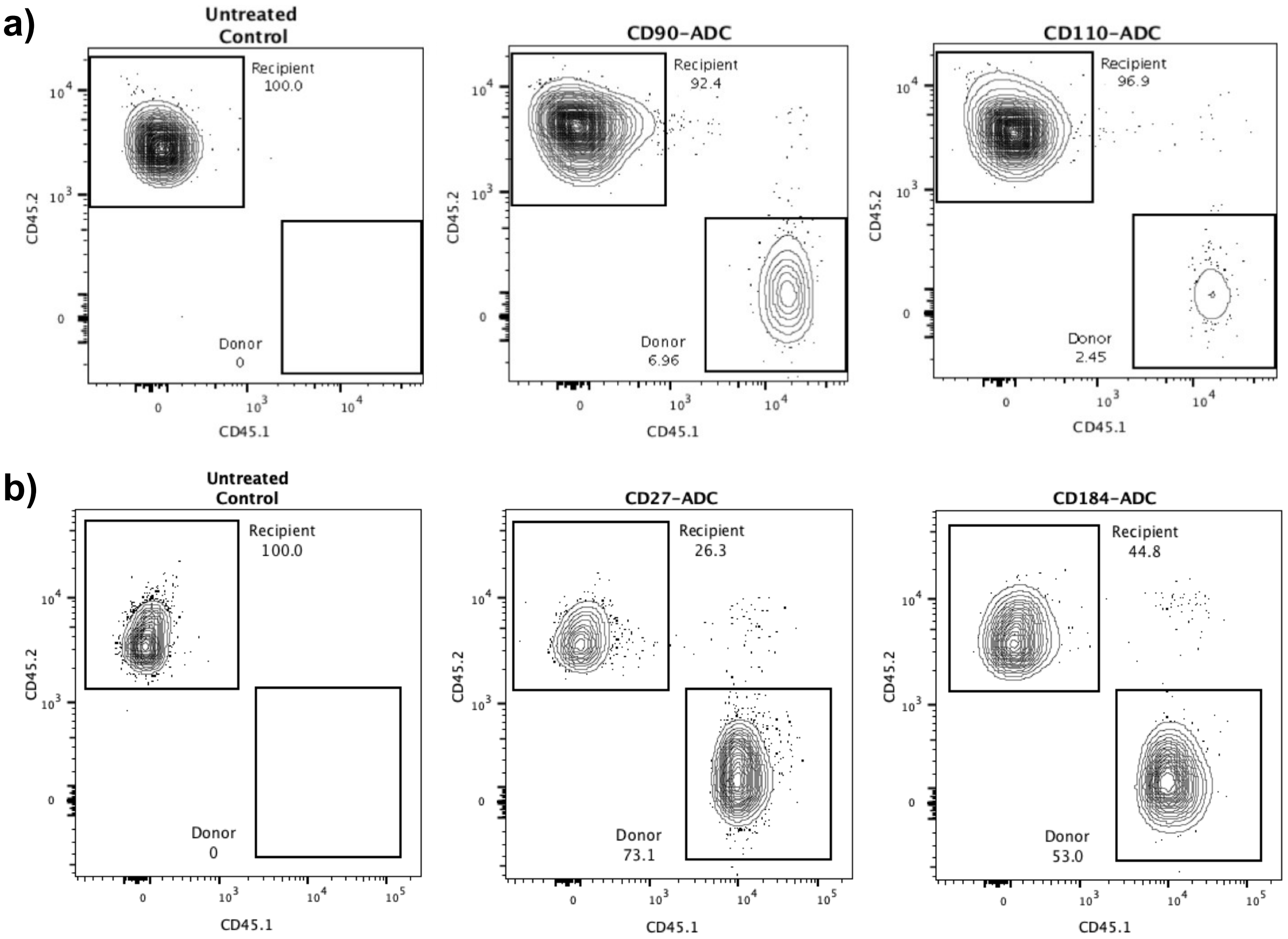

Supplementary Figure 8. Alternative specific targeting HSC-antigen ADCs also enable enhanced donor bone marrow engraftment.
Pre-treatment with HSC-ADCs (CD90-, CD110-, CD27-, and CD184-) and subsequent transplantation of 10×10^6 CD45.1+ donor whole bone marrow cells 8 days after led to enhanced donor hematopoietic cell engraftment as indicated in representative peripheral blood FACS plots (a) Exp 1 at 24 weeks and (b) Exp 2 at 12 weeks post transplantation. (n=2-4 mice/group, assayed individually).
