# Supplementary material for: Selective hematopoietic stem cell ablation using CD117-antibody-drug-conjugates enables safe and effective transplantation with immunity preservation
Source: Nat Commun. 2019 Feb 6;10:617. doi: 10.1038/s41467-018-08201-x (PMC6365495; doi:10.1038/s41467-018-08201-x)
Supplement: Supplementary file 3 — Reporting Summary [file 41467_2018_8201_MOESM3_ESM.pdf]

nature research

Corresponding author(s): Agnieszka Czechowicz

Reporting Summary

Nature Research wishes to improve the reproducibility of the work that we publish. This form provides structure for consistency and transparency in reporting. For further information on Nature Research policies, see Authors & Referees and the Editorial Policy Checklist.

Statistical parameters

When statistical analyses are reported, confirm that the following items are present in the relevant location (e.g. figure legend, table legend, main text, or Methods section).

n/a	Confirmed	
☐	☒	The exact sample size (*n*) for each experimental group/condition, given as a discrete number and unit of measurement
☐	☒	An indication of whether measurements were taken from distinct samples or whether the same sample was measured repeatedly
☐	☒	The statistical test(s) used AND whether they are one- or two-sided<br>*Only common tests should be described solely by name; describe more complex techniques in the Methods section.*
☒	☐	A description of all covariates tested
☒	☐	A description of any assumptions or corrections, such as tests of normality and adjustment for multiple comparisons
☐	☒	A full description of the statistics including central tendency (e.g. means) or other basic estimates (e.g. regression coefficient) AND variation (e.g. standard deviation) or associated estimates of uncertainty (e.g. confidence intervals)
☐	☒	For null hypothesis testing, the test statistic (e.g. *F*, *t*, *r*) with confidence intervals, effect sizes, degrees of freedom and *P* value noted<br>*Give P values as exact values whenever suitable.*
☒	☐	For Bayesian analysis, information on the choice of priors and Markov chain Monte Carlo settings
☒	☐	For hierarchical and complex designs, identification of the appropriate level for tests and full reporting of outcomes
☒	☐	Estimates of effect sizes (e.g. Cohen's *d*, Pearson's *r*), indicating how they were calculated
☐	☒	Clearly defined error bars<br>*State explicitly what error bars represent (e.g. SD, SE, CI)*

Our web collection on statistics for biologists may be useful.

Software and code

Policy information about availability of computer code

Data collection	Microsoft Excel, Graphpad Prism 7, or Treestar FlowJo was used to analyze the data as indicated in the Methods.
Data analysis	Microsoft Excel, Graphpad Prism 7, or Treestar FlowJo was used to analyze the data as indicated in the Methods.

For manuscripts utilizing custom algorithms or software that are central to the research but not yet described in published literature, software must be made available to editors/reviewers upon request. We strongly encourage code deposition in a community repository (e.g. GitHub). See the Nature Research guidelines for submitting code & software for further information.

Data

Policy information about availability of data

All manuscripts must include a data availability statement. This statement should provide the following information, where applicable:

- Accession codes, unique identifiers, or web links for publicly available datasets
- A list of figures that have associated raw data
- A description of any restrictions on data availability

The authors declare that all data supporting the findings of this study are available within the paper and its supplementary information files.

Field-specific reporting

Please select the best fit for your research. If you are not sure, read the appropriate sections before making your selection.

☒ Life sciences ☐ Behavioural & social sciences ☐ Ecological, evolutionary & environmental sciences

For a reference copy of the document with all sections, see nature.com/authors/policies/ReportingSummary-flat.pdf

Life sciences study design

All studies must disclose on these points even when the disclosure is negative.

Sample size	Sample size was usually determined by availability of animals and reagents, based upon experience with prior experiments. A majority of experiments were performed several times with at least n=3-5 biological replicates.
Data exclusions	Generally no data was excluded from analysis. Animals from in vivo experiments could have been excluded if they had unrelated health issues for which action had to be undertaken (according to institutional health policies); but generally all treatments were found to be safe and well tolerated at doses reported.
Replication	All attempts at replication were successful using reported reagents.
Randomization	Allocation of animals was random and was performed by an independent technician. He/she assigned mice to different groups in a random fashion.
Blinding	Animals were generally labeled with marking system that was subsequently unblinded after analysis. The pathologist was blinded to the treatments the animals had received for which the histology was obtained.

Reporting for specific materials, systems and methods

Materials & experimental systems

n/a	Involved in the study
☒	☐ Unique biological materials
☐	☒ Antibodies
☒	☐ Eukaryotic cell lines
☒	☐ Palaeontology
☐	☒ Animals and other organisms
☒	☐ Human research participants

Methods

n/a	Involved in the study
☒	☐ ChIP-seq
☐	☒ Flow cytometry
☒	☐ MRI-based neuroimaging

Antibodies

Antibodies used	All antibodies used in the studies are listed in the attached Methods and were commercially available for purchase.
Validation	Antibody staining was validated by observing positive and negative staining on samples; and all stains were made in batches to eliminate staining bias.

Animals and other organisms

Policy information about studies involving animals; ARRIVE guidelines recommended for reporting animal research

Laboratory animals	All mice used in the studies are listed in the attached Methods and were commercially available for purchase.
Wild animals	The study did not include wild animals.
Field-collected samples	The study did not include sample collected from the field.

Flow Cytometry

Plots

Confirm that:

- [x] The axis labels state the marker and fluorochrome used (e.g. CD4-FITC).
- [x] The axis scales are clearly visible. Include numbers along axes only for bottom left plot of group (a 'group' is an analysis of identical markers).
- [x] All plots are contour plots with outliers or pseudocolor plots.
- [x] A numerical value for number of cells or percentage (with statistics) is provided.

Methodology

Sample preparation	*Describe the sample preparation, detailing the biological source of the cells and any tissue processing steps used.*
Instrument	Flow cytometry was collected using BD FACS Aria.
Software	Flow cytometry data was collected and analyzed with FlowJo and Prism.
Cell population abundance	Isolation and abundance of cell populations in the studies are listed in the attached Methods and Supplementary Information.
Gating strategy	Gating strategy used in the studies is listed in the attached Methods and Supplementary Information.

- [x] Tick this box to confirm that a figure exemplifying the gating strategy is provided in the Supplementary Information.
